# Ovine Herpesvirus 2 Encodes a Previously Unrecognized Protein, pOv8.25, That Targets Mitochondria and Triggers Apoptotic Cell Death

**DOI:** 10.1128/JVI.01536-19

**Published:** 2020-03-31

**Authors:** Neeta Shrestha, Kurt Tobler, Stephanie Uster, Romina Sigrist-Nagy, Melanie Michaela Hierweger, Mathias Ackermann

**Affiliations:** a Institute of Virology, Vetsuisse Faculty, University of Zurich, Zurich, Switzerland; b Division of Experimental Clinical Research, Vetsuisse Faculty, University of Bern, Bern, Switzerland; c Graduate School for Cellular and Biomedical Sciences, University of Bern, Bern, Switzerland; Northwestern University

**Keywords:** malignant catarrhal fever, MCF, herpesvirus, macavirus, ovine herpesvirus 2, OvHV-2, Ov8.25, pathogenesis, apoptosis, necrosis, mitochondria, disease phenotype

## Abstract

Malignant catarrhal fever (MCF) is a rare but frequently lethal disease of certain cloven-hoofed animals. At least 10 different viruses, all members of the *Macavirus* genus in the subfamily *Gammaherpesvirinae*, are known as causative agents of MCF. Among these, ovine herpesvirus 2 (OvHV-2) is the most frequent and economically most important MCF agent. Phenotypically, MCF is characterized by severe lymphocytic arteritis-periarteritis, which leads to the accumulation of activated lymphocytes accompanied by apoptosis and necrosis in a broad range of tissues. However, a viral factor that might be responsible for tissue damage has not yet been identified. We have studied a seemingly intergenic locus on the OvHV-2 genome, which was previously shown to be transcriptionally highly active in MCF-affected tissue. We identified by 5′ and 3′ rapid amplification of cDNA ends (RACE) a conserved, double-spliced transcript that encoded a 9.9-kDa hydrophobic protein. The newly detected gene, Ov8.25, and its splicing pattern were conserved among OvHV-2 strains of different origins. Upon transient expression of synthetic variants of this gene in various cell types, including bovine lymphocytes, the protein (pOv8.25) was shown to target mitochondria, followed by caspase-dependent apoptosis and necrosis. Notably, a deletion mutant of the same protein lost these abilities. Finally, we detected pOv8.25 in brain-infiltrating lymphocytes of cattle with MCF. Thus, the cell death-causing properties of pOv8.25 in affected cells may be involved in the emergence of typical MCF-associated apoptosis and necrosis. Thus, we have identified a novel OvHV-2 protein, which might contribute to the phenotype of MCF-related lesions.

**IMPORTANCE** Ovine herpesvirus 2 (OvHV-2) circulates among sheep without causing disease. However, upon transmission to cattle, the same virus instigates a frequently lethal disease, malignant catarrhal fever (MCF). While the cause of death and pathogenesis of tissue lesions are still poorly understood, MCF is characterized by the accumulation of lymphocytes in various tissues, associated with vasculitis and cell death. As infectious virus is hardly present in these lesions, the cause of cell death cannot be explained simply by viral replication. The significance of our research is in identifying and characterizing a previously overlooked gene of OvHV-2 (Ov8.25), which is highly expressed in animals with MCF. Its encoded protein targets mitochondria, causing apoptosis and necrosis, thus contributing to an understanding of the source and nature of cell death. As the corresponding genetic locus is also active in the context of MCF due to a different macavirus, we may have detected a common denominator of the disease phenotype.

## Abstract (precis)

Ovine herpesvirus 2 (OvHV-2) circulates among sheep without causing disease. However, upon transmission to cattle, the same virus instigates a frequently lethal disease, malignant catarrhal fever (MCF). While the cause of death and pathogenesis of tissue lesions are still poorly understood, MCF is characterized by the accumulation of lymphocytes in various tissues, associated with vasculitis and cell death. As infectious virus is hardly present in these lesions, the cause of cell death cannot be explained simply by viral replication. The significance of our research is in identifying and characterizing a previously overlooked gene of OvHV-2 (Ov8.25), which is highly expressed in animals with MCF. Its encoded protein targets mitochondria, causing apoptosis and necrosis, thus contributing to an understanding of the source and nature of cell death. As the corresponding genetic locus is also active in the context of MCF due to a different macavirus, we may have detected a common denominator of the disease phenotype.

## INTRODUCTION

Malignant catarrhal fever (MCF) is a rare but frequently lethal disease affecting various species in the order *Artiodactyla* (even-toed cloven-hoofed animals). Two related viruses, alcelaphine herpesvirus 1 (AlHV-1, wildebeest-associated, SA) and ovine herpesvirus 2 (OvHV-2, sheep-associated, SA), both belonging to the *Macavirus* genus within the subfamily *Gammaherpesvirinae*, are responsible for most cases of MCF, although at least 8 additional MCF-causing viruses are known (reviewed in reference 1). The economically most important form of MCF affects cattle as well as farmed bison and deer, occurs worldwide, and is associated with OvHV-2. AlHV-1 also affects cattle but is restricted predominantly to Africa and to zoos (2–4).

Typically, there are two types of host for the same virus, a reservoir host, in which the virus circulates without causing disease symptoms, and an indicator host, which is usually free of the same virus but succumbs to MCF upon accidental infection (1, 5–9). In the case of OvHV-2, sheep are the reservoir host (therefore SA), while the main range of indicator hosts includes various members of the *Bovinae*, including cattle, water buffaloes, and bison; the *Cervidae*, including red deer and sika deer; and the *Suidae*, including the domestic pig (6, 10–17).

Various clinical forms of the disease have been described, but the so-called “head-and-eye form” is most common, with typical signs including fever, inappetence, ocular and nasal discharge, corneal opacity, mucosal lesions in the buccal and nasal cavities and around the muzzle, diarrhea, and depression. Up to 95% of affected cattle succumb to death or have to be euthanized within 1 week after the onset of the disease (6, 8, 12). Histologically, MCF is characterized by the accumulation of lymphocytes and apoptosis and necrosis in a range of tissues, but the morphological hallmark is severe lymphocytic arteritis-periarteritis with affection of the tunica media (8, 18, 19).

While viral transmission, clinical signs, and histopathological changes associated with the disease have been well characterized, major aspects of the pathogenesis of MCF are still enigmatic. It is agreed, however, that both viral and host determinants play a role. Among the viral factors involved, it has been shown at least for AlHV-1 that expression of the latency-associated nuclear antigen (LANA) plays an essential role because a LANA knockout virus did not induce MCF (20). Based on this observation, it has been concluded that the infecting virus must be able to establish a latent infection prior to the outbreak of the disease. However, the predicted properties associated with the LANA protein, which include maintenance of the viral genome during latency, cannot explain the disease phenotype. In the context of OvHV-2, such experiments have not yet been feasible because the virus cannot be propagated in conventional cell cultures, nor is an infectious bacterial artificial chromosome (BAC) clone available that would make genetic engineering of the viral genome possible. However, there is ample evidence available to suggest that the LANA gene (open reading frame 73 [ORF73]) is also expressed during MCF due to OvHV-2 (21). In addition, various reports have detected the expression of OvHV-2 lytic genes throughout the course of the disease (22, 23). It has been debated, therefore, whether lytic or latent OvHV-2 may be responsible for outbreaks of MCF. As animals suffering from MCF typically do not shed and transmit the virus, it has also been agreed that OvHV-2 replicates only poorly in the indicator animal species (1, 8, 24). Therefore, viral replication certainly plays an important role at very early stages of the infection, particularly prior to dissemination within the body. However, at later times, viral lytic genes may be transcribed and translated but certainly will not yield high viral titers. Indeed, typical herpesviral particles cannot consistently be found in affected tissues of animals with MCF but can be readily detected in nasal sheddings of seemingly healthy sheep excreting OvHV-2 (25, 26). Overall, no common denominator has yet been identified among the macaviruses that might explain the occurrence and disease phenotype of MCF.

In previous work, we detected a genetic locus within the OvHV-2 genome with high transcriptional activity in lymphatic tissues of animals with sheep-associated MCF (SA-MCF) (21). Interestingly, the same region in AlHV-1 was also transcriptionally active in tissues from animals with experimental AlHV-1-derived MCF (20). Hitherto, no gene has been allocated to this transcript, although the region in AlHV-1 has been designated E16 or ER16 (ER for expressed region), whereas we referred to the corresponding region in OvHV-2 as “intergenic” (20, 21). In the present work, we have mapped a previously overlooked viral gene, newly designated Ov8.25, to this region, noting that it gives rise to a double-spliced mRNA, which translates into a small, hydrophobic protein. Transient expression of this protein (pOv8.25) showed that it targeted the mitochondria, causing apoptosis and necrosis in transfected cells. Finally, pOv8.25 was also detected in the brain of cattle with MCF.

## RESULTS

### Sequence of a novel transcript expressed in OvHV-2-infected cells.

In previous work (21), an RNA mapping to a seemingly intergenic region of the OvHV-2 genome was detected by microarray analysis in lymph nodes of cows with MCF. Here, RNA was extracted from two different sources in order to identify the ends of the predicted molecule by 5′ rapid amplification of cDNA ends (RACE) and 3′ RACE, as described in Materials and Methods. The first RNA template originated from lymphocytes of a cow with MCF. The second RNA template was extracted from OvHV-2-infected large granular lymphocytes (LGLs).

**FIG 1:**
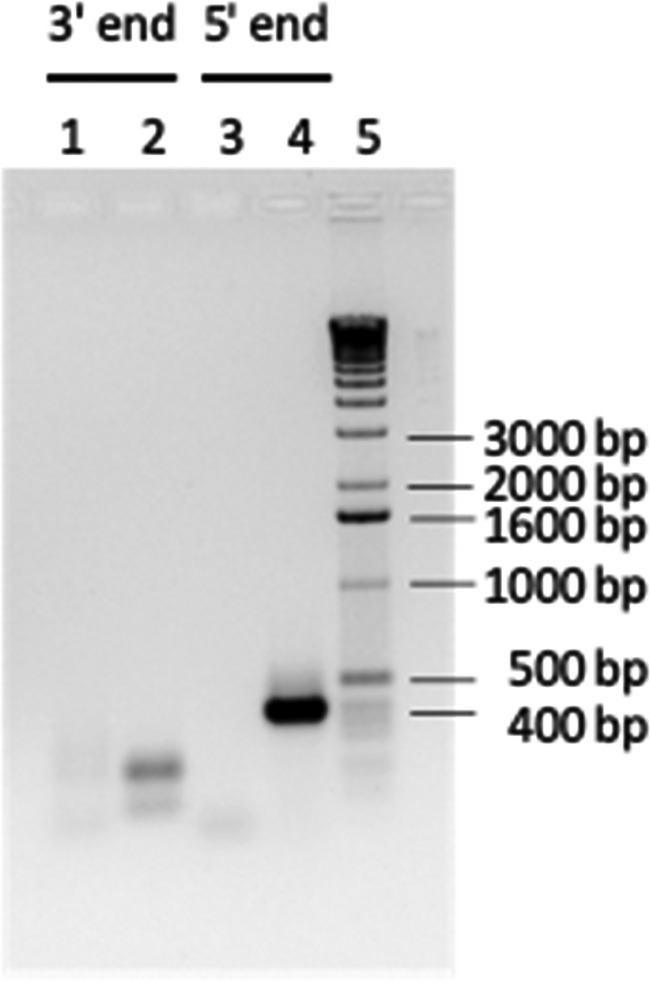
RACE experiments to determine the ends of the novel transcript. A 1% agarose gel shows RLM-RACE PCR products obtained from RNA of an MCF-affected cow. Lane 1, 3′ RACE after the first amplification; lane 2, 3′ RACE after nested amplification; lane 3, 5′ RACE after the first amplification; lane 4, 5′ RACE after nested amplification; lane 5, 1-kb DNA marker.

The products of RNA ligase-mediated RACE (RLM-RACE) PCR were loaded onto a 1% agarose gel (Fig. 1). After nested amplification, the 5′ end provided a product of approximately 400 bp (lane 4), whereas the 3′ end resulted in a 200-bp product (lane 2). Similar results were obtained with the second template (data not shown).

**FIG 2:**
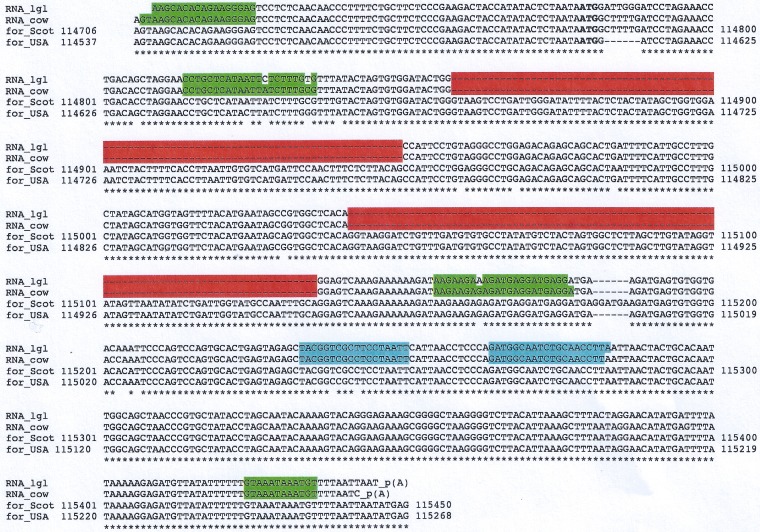
Conservation of the Ov8.25 locus and splicing. Shown is a sequence alignment of the novel transcripts found in the LGLs (RNA_lgl) and in lymphocytes of a cow with MCF (RNA_cow) against reported OvHV-2 genomic sequences. The predicted translation initiation codon (ATG) is highlighted in boldface type. The two predicted introns in the processed transcripts are highlighted in red. The asterisks indicate identical nucleotides in all four sequences. The internal gene-specific primers used for RACE are highlighted in blue. The sequences of the forward primers and the complementary sequences of the reverse primers for cloning into the pEGFP N3 expression vector are highlighted in green.

The PCR products were cloned using the TOPO TA method and submitted for sequencing. Interestingly, the sequences obtained from the two independent sources were 96% identical. The sequences were then aligned to the OvHV-2 genomes reported in GenBank (accession numbers AY839756, a Scotch strain derived from bovine LGLs, and DQ198083, an American strain derived from sheep nasal secretions). The 5′ end of the transcript from the cow with MCF mapped to position 114706 of the Scotch strain, which corresponds to position 114555 of the American strain. The 3′ end mapped to positions 115450 (Scotch strain) and 115287 (American strain). From these map positions, a transcript size of between 745 bases (Scotch strain) and 733 bases (American strain) could be predicted. However, the observed transcript size was smaller. It was also evident that there were two parts in the OvHV-2 genomes that were not detected in the transcripts. The most probable explanation for these gaps was a splicing process executed on the primary transcript. Indeed, upon *in silico* analysis of the transcript’s sequence, the NetGene2 server confirmed the observed splice sites with good statistical confidence (www.cbs.dtu.dk/services/NetGene2) (Fig. 2). Significantly, the predicted splice sites were conserved in all four sequences.

### The novel transcript is spliced upon transient expression.

**FIG 3:**
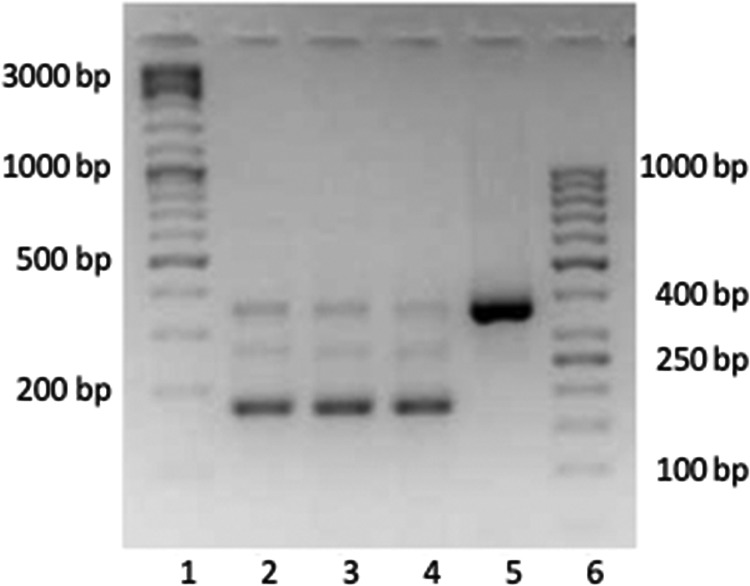
The Ov8.25 transcript is smaller than its DNA template. A 2.5% agarose gel is shown. Lanes 1 and 6, DNA size marker; lanes 2 through 4, amplified PCR products obtained from decreasing amounts of cDNA template (3.2 μl, 1.9 μl, and 1.5 μl of the cDNA template, respectively); lane 5, PCR product obtained from transfecting DNA template.

The genomic locus encompassing the two putative introns was amplified by PCR and cloned under the control of the cytomegalovirus immediate early (cmvIE) promoter into the pEGFP N3 vector for transient-expression assays. HEK 293T cells were transfected with this construct, and total RNA was harvested at 25 h posttransfection. After reverse transcription, the cDNA was PCR amplified by using primers corresponding to the terminal OvHV-2 sequences of the construct. Input DNA was used as a positive control. Figure 3 shows the results. The DNA template provided a PCR product of just below 400 bp, which corresponded well with the expected size of 364 bp (lane 5). In contrast, the cDNA template (lanes 2 through 4) provided one strong band at approximately 200 bp and two weaker bands, with the upper band migrating as the unspliced DNA template and the second migrating at an intermediate position, just below 300 bp. The strong band was consistent with the predicted size (177 bp) of the double-spliced transcript, which was verified by extracting the band from the gel followed by sequencing. The intermediate band may represent a single-spliced variant. Thus, splicing of the transiently expressed transcript took place, even in the absence of other factors contributed by OvHV-2. Based on these results, we concluded that the above-mentioned “intergenic” region of the OvHV-2 genome was by no means intergenic. Rather, it comprised a thus-far-overlooked OvHV-2 gene. BLAST analysis did not reveal similar genes in other herpesviruses. Therefore, we considered it an OvHV-2-specific gene. Due to its location on the OvHV-2 genome between ORF69 and Ov8.5, the novel gene was named Ov8.25.

### Ov8.25 encodes a protein.

**FIG 4:**
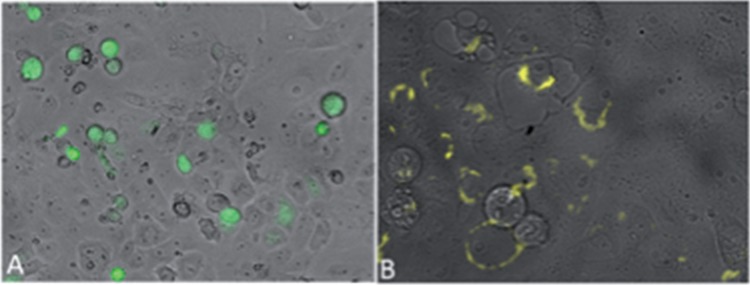
Compartmentalized localization of the pOv8.25-EYFP fusion protein. Shown are Vero 2-2 cells under a fluorescence microscope 24 h after transfection with either pHSV_EGFP (A) or pHSV_Ov8.25-EYFP (B).

To address the novel gene’s capacity to encode a protein, the above-mentioned cDNA was PCR amplified by using primers that targeted the Ov8.25 sequence beginning from the predicted ATG codon of the putative open reading frame (ORF) down to the 3′ end of the ORF but without the stop codon. This amplified sequence was then cloned into a herpes simplex virus 1 (HSV-1) amplicon vector, which also provided a C-terminal enhanced yellow fluorescent protein (EYFP) as a fusion partner for the putative Ov8.25 protein. The resulting construct was transfected into Vero 2-2 cells and analyzed under a fluorescence microscope at 24 h posttransfection. As shown in Fig. 4A, an enhanced green fluorescent protein (EGFP)-expressing control amplicon construct illuminated the body of transfected cells with green fluorescent protein (GFP). In contrast, as shown in Fig. 4B, the putative pOv8.25-EYFP fusion protein localized to the cytoplasm, predominantly around the rim of the cellular nucleus. It was noted that cells expressing the newly detected protein quite often showed an enlarged nucleus. Moreover, the yield of pOv8.25 protein seemed to be low.

### Splicing of the Ov8.25 transcript is independent of its protein-coding sequences.

**FIG 5:**
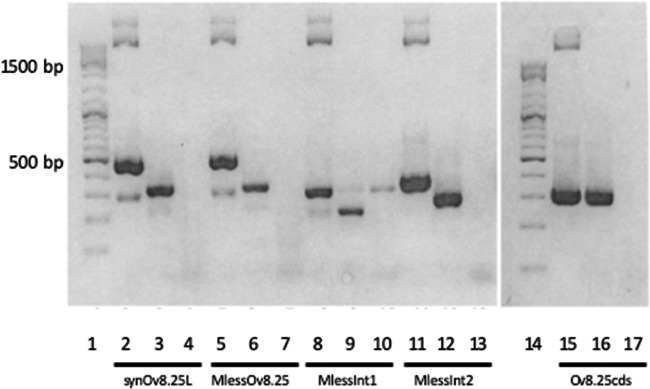
Conserved splicing of Ov8.25 transcripts. Reverse transcription-PCR was done using the RNA template harvested 24 h after transfection of Vero 2-2 cells with synthetic Ov8.25 amplicon constructs. Each construct is represented in three consecutive lanes, and each triplet is labeled beneath the lanes with the designation of the construct. The first lane (lanes 2, 5, 8, 11, and 15) in each triplet represents a control using plasmid DNA as the template. For the second lane (lanes 3, 6, 9, 12, and 16), the reverse-transcribed RNA extract was used as the PCR template. For the third lane (lanes 4, 7, 10, 13, and 17), the RNA extract was PCR amplified without prior reverse transcriptase treatment. Lanes 1 and 14, 100-bp DNA size ladder, with a strong 500-bp band.

The putative function of the Ov8.25 locus might be associated with either the encoded protein, splicing of the primary transcript, or even both features. In order to discriminate between these possibilities, synthetic constructs were generated (see Table 3). The five constructs were separately transfected into Vero 2-2 cells, and RNA was harvested after 24 h. Each RNA extract was reverse transcribed into cDNA before amplifying the sequences of interest by PCR as described in Materials and Methods. As shown in Fig. 5, the transiently expressed transcript was spliced even when the amino acid coding sequences had been optimized for translation in bovine cells (lane 3) or when protein translation was abolished after removing all start codons (ATG) from within the coding sequences and replacing them with stop codons (lane 6). The same extracts amplified without prior treatment with reverse transcriptase (lanes 4 and 7) did not yield a product. In contrast, when the corresponding plasmid DNA was used as a PCR template, a larger band became visible (lanes 2 and 5), where the size difference could be explained by the size of the introns. Similarly, the single-intron constructs were spliced, as shown in Fig. 5, lanes 9 and 12 (the corresponding controls are shown in lanes 8 and 10 and lanes 11 and 13, respectively). In contrast to all of these constructs, the synthetic intronless construct remained unspliced (lanes 15 through 17).

### Primary characterization of the pOv8.25-EYFP fusion protein.

**FIG 6:**
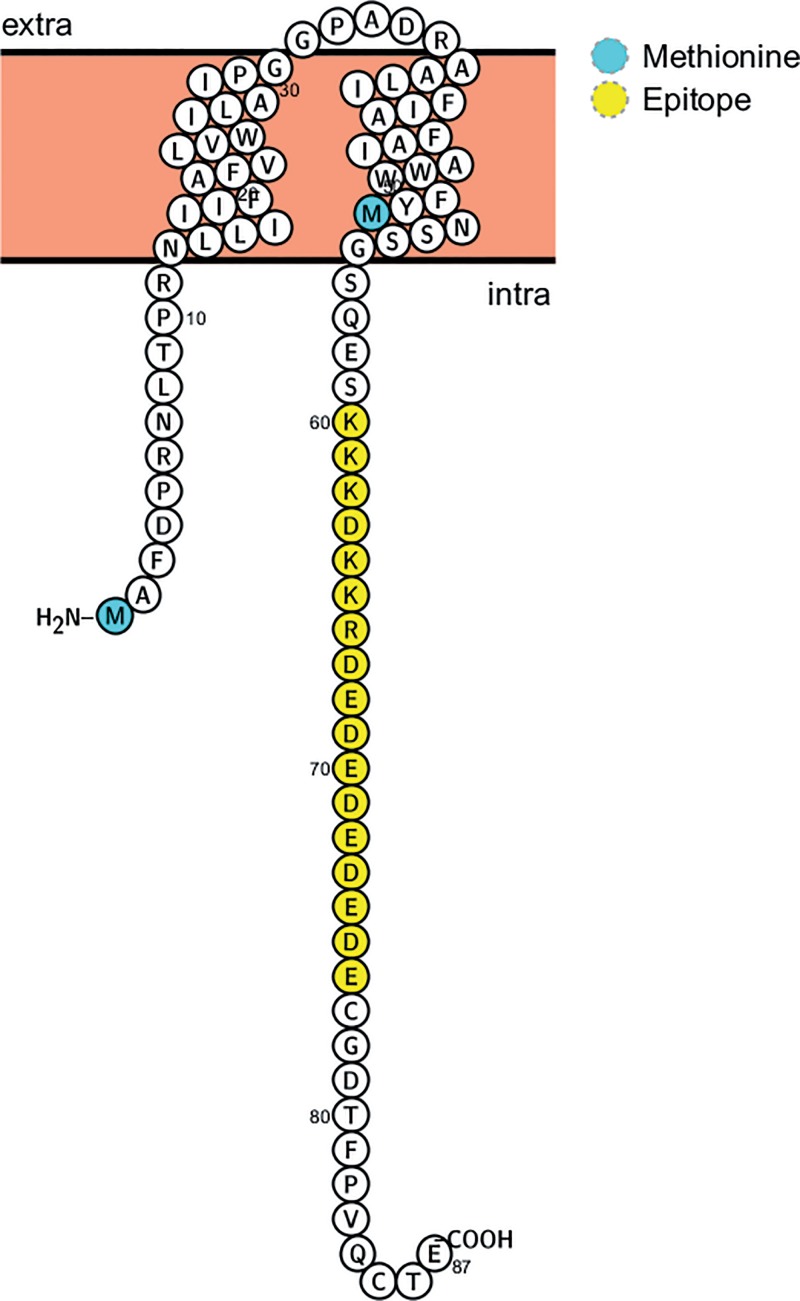
Predicted structure of pOv8.25, including M1, M51 (blue), two transmembrane domains, and a predicted antibody epitope (yellow), which was used to generate rabbit antiserum against pOv8.25. (The figure was generated using Protter [43].)

According to its amino acid sequence and although it did not feature a signal sequence, the Ov8.25 protein (pOv8.25) was predicted to be a membrane protein (Fig. 6) with two transmembrane domains and a predicted molecular weight of 9.9 kDa. In the absence of existing antibodies against pOv8.25, the encoded translation product was first characterized as an EYFP fusion protein.

**FIG 7:**
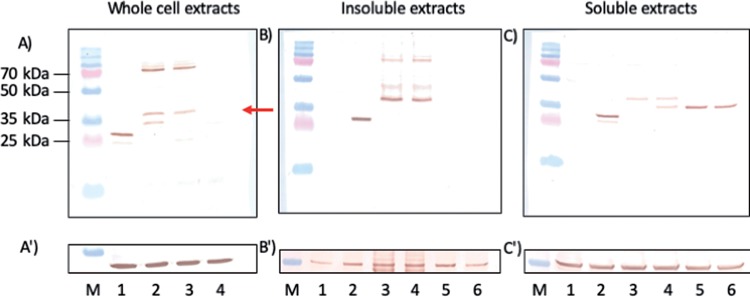
Apparent mobility and solubility of pOv8.25. (A to C) Western immunoblot of transfected Vero 2-2 cells labeled with anti-GFP monoclonal antibody. (A′) Replicate blot labeled with anti-actin monoclonal antibody. (B′ and C′) Blots from panels B and C restained with anti-tubulin monoclonal antibody. (A and A′) Lane M, molecular weight marker; blue band the left, 35 kDa; lanes 1 through 4, transfected cell extracts harvested at 24 h (lane 1, EYFP amplicon; lane 2, codon-optimized, intronless Ov8.25 coding sequence with C-terminal EYFP; lane 3, codon-optimized Ov8.25 locus with C-terminal EYFP; lane 4, untransfected cell extract). (B, B′, C, and C′) Harvested cells were suspended in IP buffer containing 1% Triton X-100 before soluble and insoluble extracts were separated by centrifugation. Both fractions were separately taken up in SDS buffer and separated by PAGE. Finally, Western transfers were stained using monoclonal antibodies. Lanes 1, nontransfected Vero cells; lanes 2 through 6, transfected Vero cells (lane 2, cells transfected with EYFP; lane 3, codon-optimized Ov8.25 locus with C-terminal EYFP; lane 4, codon-optimized, intronless Ov8.25 coding sequence with C-terminal EYFP; lane 5, 5′-truncated codon-optimized Ov8.25 locus with C-terminal EYFP; lane 6, 5′-truncated codon-optimized, intronless Ov8.25 coding sequence with C-terminal EYFP). The red arrow indicates 38.7 kDa, the predicted mobility of the pOv8.25-EYFP fusion protein.

To this end, Vero 2-2 cells were transfected with amplicon vectors encoding either EYFP alone or EYFP as a C-terminal fusion to pOv8.25. Twenty-four hours after transfection, yellow fluorescence was observed in approximately 50% of the cells, which were then harvested for analysis by Western immunoblotting using a monoclonal antibody (mcAb) against GFP (Fig. 7A). EYFP was expected to migrate at approximately 26.9 kDa, and the pOv8.25-EYFP fusion protein was expected to migrate at 38.8 kDa. However, a second, faster-migrating band of approximately 33 kDa was visible in lane 2 but absent in lane 3. The size of this 33-kDa band corresponds well to the predicted molecular weight of an N-terminally truncated Ov8.25-EYFP fusion protein, whose translation would start only at the second methionine codon (position M51). Such a truncation would remove the predicted transmembrane domains of pOv8.25, which might interfere with its subcellular localization and biological function. Interestingly, slower-migrating bands at about 70 kDa were also stained by the anti-GFP antibody in each of the two Ov8.25-transfected extracts in lanes 2 and 3 (Fig. 7A). The same extra bands were also visible in the insoluble extract (Fig. 7B), where even an additional 48-kDa band emerged. These higher-molecular-weight bands were not visible in the EYFP extract, the nontransfected cell extract (lane 4), or the soluble cell extracts (Fig. 7C). The nature of these bands cannot yet be rationally explained, yet potential reasons may include dimerization or covalent binding to a host protein.

In order to address the nature of the 33-kDa band that was visible only in extracts from cells transfected with the codon-optimized, intronless Ov8.25 coding sequence, two 5′ deletion mutants were constructed, one yielding a 5′-truncated, codon-optimized Ov8.25 locus with C-terminal EYFP and the other giving a 5′-truncated, codon-optimized, intronless Ov8.25 coding sequence with C-terminal EYFP. Both constructs had their translation initiation codon at the above-described position 51 (M51) (Fig. 6), which caused a loss of the membrane anchoring of the truncated protein. Accordingly, we hypothesized that the truncated proteins may be more soluble than their original counterparts. To address this issue, cells were transfected with either the original constructs or their truncated counterparts. Nontransfected cells and cells transfected with the EYFP amplicon served as controls. After harvesting, the cells were suspended in a buffer containing Triton X-100 before soluble and insoluble components were separated by centrifugation. Soluble and insoluble extracts were separated by SDS-PAGE, transferred to Western blots, and stained using the anti-GFP mcAb. The results are shown in Fig. 7B and C. By itself, EYFP divided almost uniformly into soluble and insoluble fractions (Fig. 7B and C, lanes 2). However, a fainter and faster-migrating band was additionally observed in the soluble fraction (Fig. 7C, lane 2). In contrast, the full-length Ov8.25 fusion proteins accumulated predominantly in the insoluble fraction (Fig. 7B, lanes 3 and 4). Indeed, the high-molecular-weight bands were readily detected in the insoluble fractions but invisible in the soluble fractions (Fig. 7C, lanes 3 and 4). However, the predicted 38.8-kDa band was discernible in both fractions. Interestingly, the M51-truncated proteins were not detected in the insoluble fractions (Fig. 7B, lanes 5 and 6) but were readily discernible among the soluble proteins (Fig. 7C, lanes 5 and 6). The 33-kDa protein produced in cells transfected with the codon-optimized, intronless Ov8.25 coding sequence was not seen among the insoluble proteins (Fig. 7B, lane 4) but sided entirely with the soluble fraction (Fig. 7C, lane 4). Moreover, it migrated at the same velocity as the truncated versions of pOv8.25, which was consistent with the hypothesis that it actually represented a secondary translation product of the codon-optimized fusion protein, which used M51 as its start codon. Since actin, shown in Fig. 7A′, migrated very close to the fusion proteins of interest, we used the slower-migrating tubulin as a loading control. The blots in Fig. 7B and C were restained using a monoclonal antibody against tubulin. The immunoblots in Fig. 7B′ and Fig. 7C′ clearly demonstrate that a major part of tubulin went into the soluble fraction, although enough tubulin was retained in the insoluble fraction to show that comparable amounts of cell extracts had been loaded into each lane.

### Identification of the Ov8.25 protein by antibodies to a synthetic peptide.

**FIG 8:**
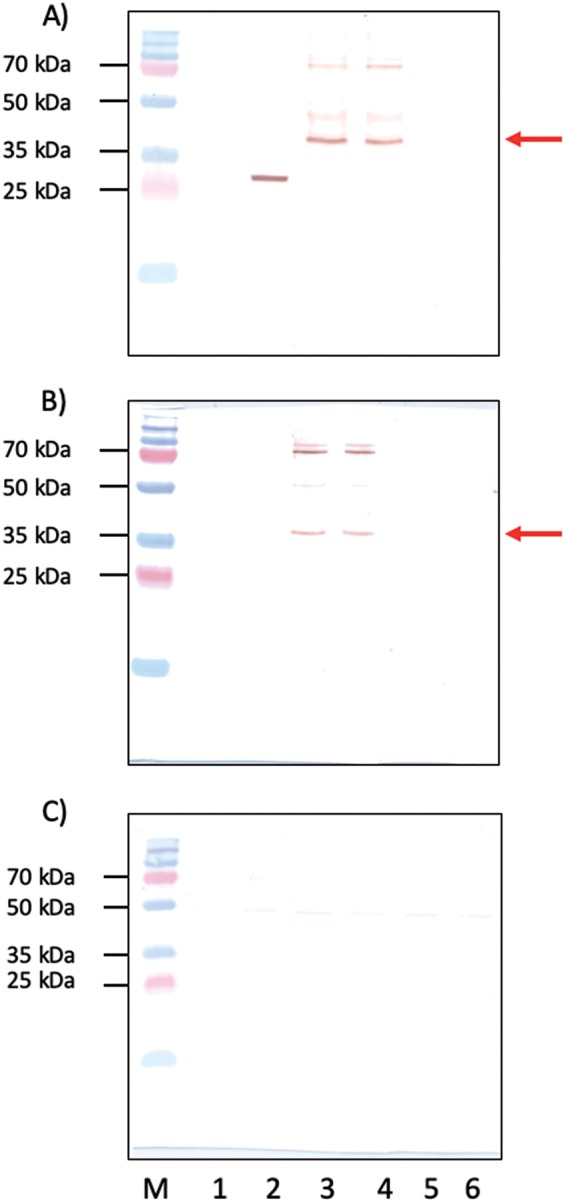
Identification of pOv8.25 by antipeptide serum. Shown are Western immunoblots of insoluble cell extracts labeled with anti-GFP monoclonal antibody (A), with rabbit anti-pOv8.25 serum (B), and with an antibody that was primarily saturated with an immunizing peptide (C). Lane M, PAGE ruler plus molecular weight marker (molecular weights are indicated on the left); lane 1, nontransfected Vero cells; lane 2, cells transfected with EYFP; lane 3, cells transfected with the codon-optimized Ov8.25 locus with C-terminal EYFP; lane 4, cells transfected with the codon-optimized, intronless Ov8.25 coding sequence with C-terminal EYFP; lane 5, truncated version of the codon-optimized Ov8.25 locus with C-terminal EYFP; lane 6, truncated version of the codon-optimized, intronless Ov8.25 coding sequence with C-terminal EYFP. Red arrows in panels A and B point to 38.7 kDa, the predicted mobility of the pOv8.25-EYFP fusion protein. Note that the higher-molecular-weight bands are recognized by both rabbit antipeptide serum and the anti-GFP mcAb. The EYFP band (lane 2) is not recognized by rabbit antipeptide serum.

To open the possibility of recognizing the Ov8.25 protein in its native form, without a tag, rabbits were immunized with a synthetic peptide that had been predicted to represent an immunogenic part of pOv8.25, namely, spanning amino acids (aa) 60 to 76 of the predicted protein (Fig. 6). Vero cells were transfected with either the EYFP amplicon, the codon-optimized, intronless Ov8.25 coding sequence with C-terminal EYFP, or the codon-optimized Ov8.25 locus with C-terminal EYFP, or, as a negative control, they were left untransfected. Furthermore, the truncated versions of the two Ov8.25 fusion proteins were also included in the experiment. After 24 h, the cells were harvested, and the insoluble fractions were subjected to SDS-PAGE and Western transfer. Identical blots were immunostained with either rabbit anti-pOv8.25 peptide serum or anti-GFP mcAb. The results are shown in Fig. 8. As expected, the rabbit antipeptide serum (Fig. 8B) was able to stain the predicted 38.7-kDa pOv8.25-EYFP fusion proteins (lanes 3 and 4) but did not react with EYFP alone (lane 2) or with nontransfected Vero cells (lane 1). As expected, the truncated forms of pOv8.25 were not detected on the blot since they had been removed with the soluble fractions.

Moreover, the rabbit antipeptide serum also recognized the higher-molecular-weight bands in Fig. 8B, lanes 3 and 4, migrating just above 70 kDa. Bands of much the same sizes were also recognized by the anti-GFP mcAb in Fig. 8A, lanes 3 and 4. Importantly, the rabbit antipeptide serum no longer reacted with the same proteins if it was saturated with the immunizing peptide (Fig. 8C). Together, these data suggest that the antipeptide serum actually recognized the pOv8.25 fragment of the fusion protein and that this protein existed in at least two forms in transfected cells, namely, in a monomeric form with the expected size of 38.7 kDa and in higher-molecular-weight forms of >70 kDa.

### The pOv8.25-EYFP fusion protein targets mitochondria.

As the pOv8.25 protein had been observed to assume a cytoplasmic localization (Fig. 4), it was of interest to know where exactly this could be. Analysis of its amino acid sequence (Fig. 6) revealed two potential transmembrane domains (aa 13 to 32 and aa 37 to 56). Moreover, a binding-protein-dependent transport system 1 had been predicted for aa 9 to 31. All of this *in silico* information suggested that pOv8.25 may represent a membrane protein, although a classical signal sequence was not identified.

**FIG 9:**
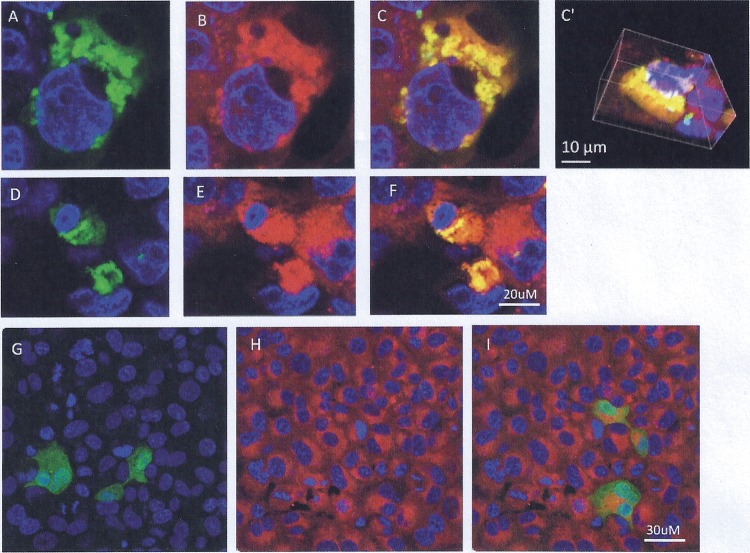
pOv8.25-EYFP colocalizes with the MitoTracker stain. Vero cells were transfected with either Ov8.25-EYFP amplicon DNA (A through F) or a truncated version that is translated only from the ATG codon encoding M51 of the original amino acid sequence (G through I). The cells were fixed at 24 h before being stained with DAPI and analyzed by confocal microscopy. (A, D, and G) Yellow fluorescence and DAPI. (B, E, and H) MitoTracker stain and DAPI. (C, C′, F, and I) Colocalization of EYFP and red fluorescence (MitoTracker), indicated by yellow. Panel C′ shows a three-dimensional reconstruction of the cell presented in panel C.

Confocal microscopy was used to address this issue. Vero cells were transfected with Ov8.25-EYFP amplicon DNA. Separate samples were harvested at 6, 24, and 48 h posttransfection and stained with fluorescent markers for individual membranous compartments, i.e., MitoTracker to test colocalization with mitochondrial membranes, concanavalin A (ConA) to make the endoplasmic reticulum (ER) compartment visible, and wheat germ agglutinin (WGA) to visualize the Golgi membranes. As shown in Fig. 9, pOv8.25-EYFP (Fig. 9A, C, D, and F) colocalized with the MitoTracker stain (Fig. 9B, C, E, and F), which was corroborated by reconstructing the three-dimensional structure of the cell (Fig. 9C′). As a control, an N-terminally truncated version of pOv8.25 (TOv8.25CDS-EYFP) (starting at M51 [Fig. 6]) was analyzed in the same manner but did not colocalize with a particular subcellular structure (Fig. 9G and I). Thus, intact pOv8.25 was required to achieve the particular subcellular localization of the fusion protein.

**FIG 10:**
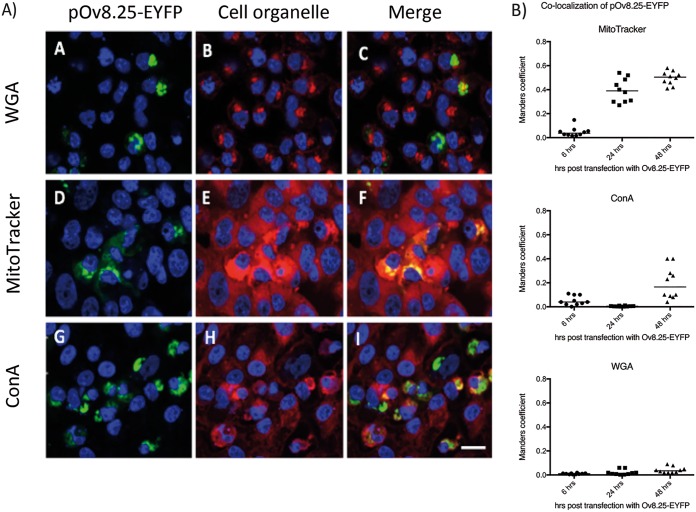
The pOv8.25-EYFP fusion protein colocalizes predominantly with mitochondria. (A) Confocal images supplemented with DAPI stain to show cell nuclei (blue). Membrane staining is seen in red, yellow fluorescence appears in green, and colocalization appears in yellow. Vero cells were transfected with Ov8.25-EYFP amplicon DNA and stained with WGA (panels B and C), red-fluorescent MitoTracker stain (panels E and F), and ConA (panels H and I) in order to test the colocalization of pOv8.25-EYFP with individual membrane compartments. Bar, 30 μm. (B) Quantitative analysis of colocalization at 6, 24, and 48 h posttransfection (determination of Manders coefficient as described in Materials and Methods).

Moreover, the results shown in Fig. 10 indicated that pOv8.25-EYFP indeed colocalized predominantly but not exclusively with mitochondria. At 48 h, a significant colocalization with ER membranes was also noted. In contrast, colocalization with Golgi membranes was not detected under the present experimental conditions.

### The pOv8.25-EYFP fusion protein induces cell death in a bovine lymphocyte cell line.

**FIG 11:**
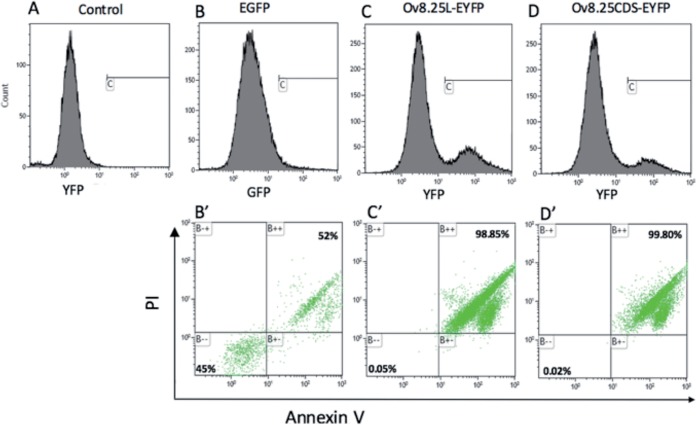
Synthesis of pOv8.25 is associated with increased cell death in a bovine Treg-like cell line. Tp951-f53 cells were left nontransduced (A) or were transduced with either EGFP (B and B′) or Ov8.25 constructs (Ov8.25L-EYFP [C and C′] or Ov8.25CDS-EYFP [D and D′]). After 24 h, the cells were harvested, double stained with annexin V and propidium iodide, and gated for fluorescence due to GFP or EYFP. Panels A to D show the total cells, where gate C shows the population that has been gated for EYFP expression. Panels B′ to D′ show AV/PI staining of the gated population.

The above-described observations that cells expressing the newly detected protein quite often showed an enlarged nucleus and that the yields of pOv8.25-EYFP protein always seemed to be low obtained a new significance when colocalization of the protein with the mitochondrial membranes was observed. Accordingly, we hypothesized that pOv8.25 may induce cell death. To test this hypothesis and to see whether or not this was the case in bovine lymphocytes, we made use of Tp951-f53, a previously characterized Theileria parva-transformed bovine cell line (27), which was triple positive for CD4, CD25, and FoxP3, thus carrying the markers of regulatory T cells (Tregs). Accordingly, Tp951-f53 cells were nontransduced or transduced with either Ov8.25L-EYFP, Ov8.25CDS-EYFP, or, as a negative control, a GFP construct. After 24 h, the cells were stained with annexin V (AV) and propidium iodide (PI), and successfully transduced cells were gated according to their fluorescence before analysis of their status concerning AV and PI by fluorescence-activated cell sorter (FACS) analysis. The results are shown in Fig. 11. Nontransduced cells (Fig. 11A) were used to set the cutoff for fluorescing cells in gate C. Although the transfection rate was not high, fluorescent cells could be detected in Fig. 11B through D. These fluorescent cells in gate C were analyzed for their AV and PI status. As shown in Fig. 11B′ 45% of the GFP-transduced cells were double negative for AV and PI, whereas 52% were double positive. However, upon transduction with the Ov8.25 constructs, the ratio of double-positive cells amounted to nearly 99%, with only a few cells remaining viable (Fig. 11C′ and D′).

We concluded from these observations that, indeed, pOv8.25 may induce cell death, even in an immortalized bovine lymphocyte cell line.

### Investigation into the pathway of pOv8.25-mediated cell death.

**FIG 12:**
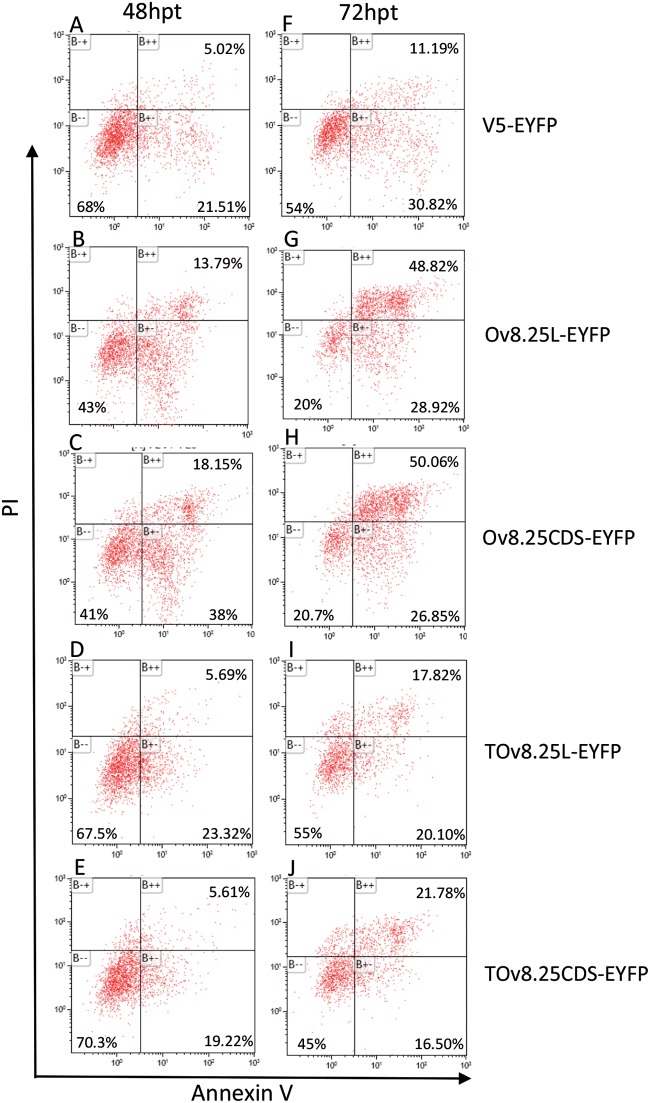
Flow cytometry analysis of AV-Cy5/PI-double-stained and EYFP-gated cells. Vero 2-2 cells were transfected with either V5-EYFP (A and F), Ov8.25L-EYFP (B and G), Ov8.25CDS-EYFP (C and H), TOv8.25-EYFP (D and I), or TOv8.26CDS-EYFP (E and J). Cells harvested at 48 and 72 h posttransfection (hpt) were labeled with AV-Cy5 and PI and analyzed by flow cytometry. Cells gated for EYFP expression are shown. AV negative/PI negative (B–), viable cells; AV positive/PI negative (B+–), apoptotic cells; AV positive/PI positive (B++), late apoptotic/dead cells.

In order to address the pathway of pOv8.25-mediated cell death, Vero 2-2 cells were transfected with Ov8.25-EYFP constructs or, as controls, with the N-terminally truncated Ov8.25-EYFP constructs or EYFP alone. Replicate cultures were harvested at 48 and 72 h posttransfection and double stained with AV and PI prior to FACS analysis. The results showing EYFP-gated cells are presented in Fig. 12.

Upon transfection, cell viability decreased over time, even with the three control constructs (EYFP, TOv8.25-EYFP, and TOv8.25CDS-EYFP). However, 48 h after transfection with these controls, 67 to 70% of the cells remained viable. In contrast, only 41 to 43% of the cells transfected with intact Ov8.25-EYFP remained viable. At 72 h, the number of viable cells was further reduced, with 45 to 55% remaining viable upon transfection with the control constructs but only 20% of cells surviving upon transfection with intact Ov8.25. Moreover, the proportion of annexin V-positive but PI-negative cells at 48 h posttransfection amounted to approximately 40% for cells transfected with intact Ov8.25, whereas only around 20% of the controls were positive for this marker of apoptosis. At 72 h posttransfection, the proportion of annexin V-positive but PI-negative cells remained almost stable for the controls (20 to 30%), while it actually decreased for the cells transfected with intact Ov8.25 (26 to 29%). In contrast, the proportion of double-positive cells was 2- to 3-fold higher in cells transfected with intact Ov8.25 (48 to 50%) than in the controls (11 to 21%).

Thus, cells transfected with intact Ov8.25 went into apoptosis (AV positive/PI negative) at around 48 h posttransfection and showed predominantly signs of late apoptosis (AV and PI double positive) at 72 h posttransfection.

### pOv8.25-mediated cell death is inhibited by caspase inhibitors.

**FIG 13:**
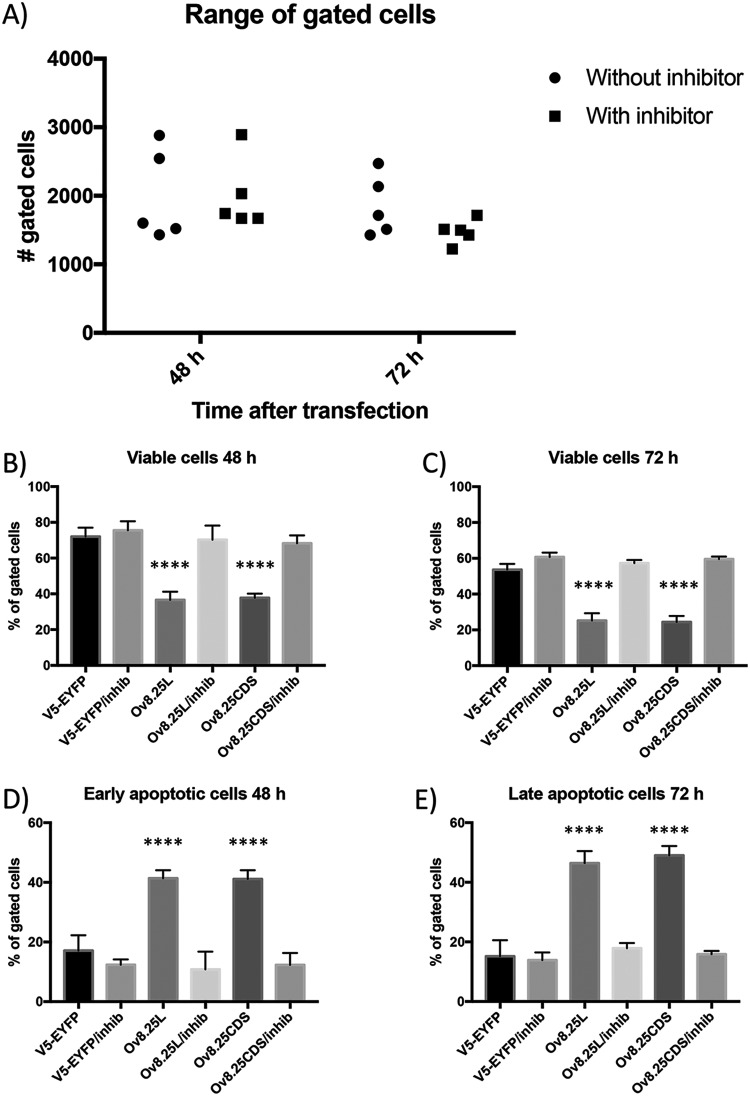
Caspase-dependent cell death upon transient expression of Ov8.25. Vero cells were transfected with constructs encoding Ov8.25-EYFP fusion proteins or EYFP alone and incubated in either the presence or absence of a broad-spectrum caspase inhibitor. The cells were harvested after 48 and 72 h, respectively, and stained for AV and PI before being gated for yellow fluorescence. (A) Counts of EYFP-positive, gated cells in each of the five experiments are shown separately for cells that had been incubated in the absence or presence of the inhibitors as well as for 48 h or 72 h posttransfection. (B and C) Median values and standard deviations from five independent experiments depicting the proportions of viable (AV/PI-double-negative) cells at 48 h (B) or 72 h (C). (D) Proportions of early apoptotic cells at 48 h (AV positive/PI negative). (E) Proportions of late apoptotic cells at 72 h (AV/PI double positive). Each column in a panel, representing replicate values from five independent experiments, was compared individually to each other column in the same panel. Significant differences were detected using the unpaired *t* test (**** indicates a *P* value of <0.0001).

Having established that pOv8.25 targeted mitochondria and that successfully transfected cells showed consecutive signs of early and late apoptosis, it was of interest to test whether or not this effect could be inhibited by caspase inhibitors. Similar to the above-described experiment, Vero 2-2 cells were transfected with Ov8.25-EYFP constructs or, as a negative control, with EYFP alone. Replicate cultures were incubated in either the presence or the absence of a pancaspase inhibitor, Z-VAD-FMK [*N*-benzyloxycarbonyl-Val-Ala-Asp(O-Me) fluoromethyl ketone]. Replicate cell cultures were harvested at 48 and 72 h posttransfection and double stained with AV and PI prior to FACS analysis. The experiment was repeated five times and, despite various transfection efficiencies (Fig. 13A), always with the same outcome, namely, increased cell death in the presence of the Ov8.25 protein (Fig. 13B through E). At both 48 and 72 h, the proportion of live cells (AV/PI double negative) among Ov8.25-transfected cells was significantly reduced compared to that among EYFP-transfected cells (Fig. 13B and C). However, in the presence of caspase inhibitors, the viabilities of Ov8.25- and EYFP-transfected cells were not significantly different. Interestingly, the predominant phenotype of nonviable cells at 48 h differed from that at 72 h. At 48 h, the proportion of early apoptotic cells (AV positive/PI negative) was significantly increased among Ov8.25-transfected cells without caspase inhibitors compared to either EYFP-transfected cells, independent of caspase inhibitors, or Ov8.25-transfected cells in the presence of caspase inhibitors (Fig. 13D). However, at 72 h, late apoptotic cells (AV/PI double positive) showed a similar, highly significant difference between Ov8.25-transfected cells without and Ov8.25-transfected cells with caspase inhibitors or EYFP-transfected cells, independent of the presence or absence of inhibitors (Fig. 13E). A general loss of viable cells observed at 72 h compared to 48 h posttransfection was attributed to stressing the cells due to the transfection protocol.

Thus, the cell death induced following the transient expression of pOv8.25 was indeed due to caspase-dependent cell death.

### Detection of pOv8.25 in brain-infiltrating lymphocytes of cattle with MCF.

**FIG 14:**
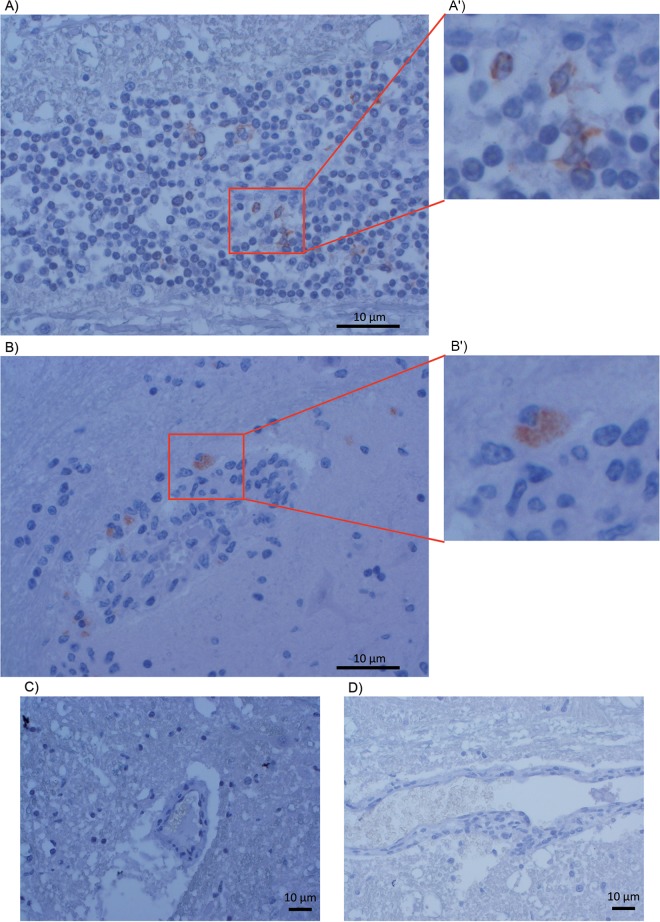
Detection of pOv8.25 in MCF-like lesions. Sections of brain tissues from cattle with encephalitis were immunostained with rabbit anti-pOv8.25 peptide serum. (A and B) Positive reactions showing reddish coloration due to the AEC chromogen in two independent cases, one of which had previously been deemed positive for OvHV-2 by PCR. (A′ and B′) Magnified areas of typical staining. (C and D) Samples from two additional unrelated cases that did not show positive reactions and that had previously been deemed negative for OvHV-2 by PCR.

Based on the above-described results, it was of interest to test whether or not pOv8.25 was detectable in pathognomonic lesions of cattle with MCF. Due to the scarcity of new cases, historical samples of cattle with histologically confirmed encephalitis were used for this purpose. Indeed, immunohistological signals of pOv8.25 were detected among infiltrating lymphocytes in brain sections of two of these cases (Fig. 14A and B). The same signals remained undetectable upon prior saturation of the antiserum with the immunizing peptide. Although they comprised histologically similar brain lesions, the other two cases, which had previously been deemed OvHV-2 negative by PCR, did not show any detectable pOv8.25 (Fig. 14C and D). Thus, as a proof of principle, pOv8.25 could be detected within typical MCF lesions (case 44282).

## DISCUSSION

In this communication, we describe a previously overlooked gene of OvHV-2, which was noticed only because of its abundant expression in animals with MCF (21). In lymphatic tissues of such animals as well as in persistently infected cell cultures (LGLs) and upon transient expression in transfected or transduced cells, this newly identified gene is expressed as a double-spliced mRNA. This gene, termed Ov8.25 due to its location within the OvHV-2 genome, encodes a protein (pOv8.25) that targets mitochondria in a manner to induce apoptosis and necrosis.

There are several distinct viruses that cause clinically and histopathologically very similar illnesses, all of which are believed to represent the same disease, MCF. These viruses are genetically similar to each other and taxonomically grouped together in the genus *Macavirus* of the subfamily *Gammaherpesvirinae*, yet each one of them also has a number of specific genes that discriminate these viruses as a group from the other genera of the gammaherpesviruses and, at the individual level, each member from the other members of the genus (1, 21, 28). These genes are named in relation to the virus to which they belong, for example, A1 through A10 in the case of AlHV-1 or Ov2 through Ov10 in the case of OvHV-2. The nomenclature stems from the sequence of AlHV-1, which was published first and therefore is recognized as the prototype (29). The numbering was from left to right in the original sequence. Later on, additional members of these specific genes were detected, which led to the introduction of “0.5” nomenclature, depending on the relative position of the newly detected gene in the original enumeration (8, 30, 31). For example, A4.5 was designated such for its position between A4 and A5. The Ov nomenclature was then adopted to use the same enumeration for potential orthologues of particular A genes in OvHV-2 (30, 31). For example, a potential orthologue of A1 was not detected in the OvHV-2 sequence. Consequently, OvHV-2 has no Ov1. However, the first OvHV-2-specific gene was termed Ov2 for its similarity to A2. Moreover, the OvHV-2 genome has an additional specific gene between Ov2 and Ov3, which was designated Ov2.5 (32). Based on these considerations and the map location of the newly detected gene between Ov8 or, more narrowly, ORF69 and Ov8.5, we named it Ov8.25. Interestingly, the Ov8.25 gene not only is conserved among various OvHV-2 isolates (Fig. 2) but also has a positional homologue in AlHV-1, termed expressed region 16 (ER16), which is highly transcribed during experimental MCF (20). Of note, a small open reading frame within ER16 has the capacity to encode an 11.3-kDa hydrophobic protein with two transmembrane regions, which shares a PVQC motif near its C terminus with our newly detected pOv8.25. Thus, this genomic locus and its protein may be some of the important players in the pathogenesis of MCF, which, despite recent progress, is still not fully understood (8, 21, 33–36). However, it remains to be confirmed that an orthologue of this gene also exists in other macaviruses.

Based on recent evidence, it can be assumed that the main portal of entry of OvHV-2 into cattle is by the respiratory route and relies on at least a few rounds of lytic replication of OvHV-2 in deep lung tissue (23, 37). Later on, OvHV-2, as well as AlHV-1, is disseminated systemically in the infected organism, affecting different types of peripheral blood mononuclear cells (PBMCs), particularly lymphocytes, but remaining in a predominantly latent form of infection as evidenced by viral gene expression assays (20, 21, 38). With the onset of clinical symptoms, a loss of overall lymphocyte counts goes along with the histological picture of nonpurulent inflammation, apoptosis, and necrosis of not only lymphoid tissue but also blood vessels in various organs due to the accumulation of activated T cells (8). Our finding that pOv8.25 targets mitochondria to induce apoptosis and necrosis fits in very well with these histopathological findings in animals with MCF. The detection of pOv8.25 in infiltrating lymphocytes within typical MCF lesions (Fig. 14) supports this notion as a proof of principle.

In the light of our present findings, we speculate that particularly the pronecrotic property of pOv8.25 (Fig. 12) might contribute to the severity and perpetuation of the disease: the expression of pOv8.25 in various cell types, thus causing their apoptosis and necrosis, will facilitate extravasation of PBMCs, and since they are also infected and expressing Ov8.25, the generation of necrotic foci around such vessels might eventually attract an inflammatory reaction. In this context, it was important to show that a truncated form of pOv8.25 had lost its ability to target mitochondria (Fig. 9) and, consequently, failed in its pronecrotic ability (Fig. 12). Moreover, we were able to demonstrate that the killing activity of pOv8.25 extends even to seemingly immortalized bovine lymphocytes with a Treg phenotype (Fig. 11), whose regulating activity would be much needed to curb the destructive effects of activated killer cells. Our results also shed new light on the old debate about the roles of latent or proliferative forms of the virus throughout the disease. For AlHV-1, it has been firmly established that an intact ORF73, encoding the major latency-associated nuclear antigen (LANA), is essential for the development of wildebeest-associated MCF (WA-MCF) (20). It has not been possible to establish the same for OvHV-2 and SA-MCF, because due to its inability to replicate in conventional cell cultures, there has not yet been any success in genetically modifying OvHV-2. However, a less strictly regulated gene expression pattern during MCF has been observed for OvHV-2 than for AlHV-1 (reviewed in reference 1). Indeed, transcripts of several lytic OvHV-2 genes have consistently been observed in MCF lesions, including, among others, the transcript for the major capsid protein, which is encoded by ORF25 (23). In the rabbit model, the lytic proteins encoded by ORF43 and ORF67 could be detected directly within MCF lesions by using specific antisera (22). However, these observations are in contrast to the absence of demonstrable viral particles in the lesions of clinically affected animals (1, 39, 40).

### Unresolved issues.

Unresolved issues are as follows. (i) From our Western blots (Fig. 7), we gained the suspicion that pOv8.25 either interacts covalently with at least one host protein or is able to form homopolymers. It was beyond the scope of this work to address this issue further, but it would be highly interesting to know the putative interaction partners of pOv8.25. (ii) It is well accepted that viruses do not carry genes for no purpose. Therefore, we have to assume that the Ov8.25 locus, its protein, as well as potentially its introns play a role in the natural life cycle of OvHV-2 among sheep. Understanding this role would be very helpful for considering what goes wrong to cause MCF in the indicator host. It might be possible to address this issue in the future, provided that a BAC-cloned OvHV-2 would be available. (iii) As we recognize that Ov8.25 seems to be important enough for OvHV-2 to keep it conserved among vastly different isolates (Scotch, American, and Swiss) (Fig. 2), we also have to assume that it is expressed throughout replication and/or latency in sheep, yet its true function remains obscure.

### Conclusions.

We have characterized a previously overlooked genetic locus in OvHV-2, which is conserved among viruses causing malignant catarrhal fever and highly transcribed throughout disease. In OvHV-2, the transcript is double spliced, giving rise to a short protein with two transmembrane regions, which targets mitochondria, causing apoptosis and necrosis in various cell types, and which can be detected in affected brain tissue. An N-terminal truncation of this protein abrogates its proapoptotic properties. With Ov8.25 mRNA (21) and its encoded protein, with its newly reported properties, present in MCF lesions, we may now begin to understand some destructive aspects of the disease phenotype. We even speculate that pOv8.25 might represent an important trigger for the development of MCF.

## MATERIALS AND METHODS

### Ethics statement and animals.

The study did not require anesthesia, euthanasia, or any kind of animal sacrifice.

### Samples from animals with MCF.

Lymphocytes from a Swiss cow with OvHV-2-associated MCF were kindly provided by Marco Franchini (University of Zurich, Switzerland).

### OvHV-2-infected large granular lymphocytes.

Persistently OvHV-2-positive large granular lymphocytes (LGLs) were kindly provided by George Russel, Moredun Research Institute, Edinburgh, Scotland.

### RLM-RACE and cloning.

**(i) RLM-RACE.** Total RNA was extracted from OvHV-2-positive materials, and 2 to 5 μg was used for RNA ligase-mediated rapid amplification of cDNA ends (RLM-RACE) (Ambion FirstChoice RLM-RACE kit’ Thermo Fisher Scientific). The protocol was performed essentially according to the supplier’s recommendations. Briefly, for 5′ RACE, RACE adapters were ligated to the targeted RNA templates before reverse transcription was initiated using a sequence-specific reverse primer. The cDNA was then used for nested PCR. For 3′ RACE, reverse transcription was started from the 3′ RACE adapter, whereas the consecutive nested PCR needed a pair of sequence-specific forward primers, bracketing the targeted sequence with a pair of reverse primers provided in the kit. Eventually, these PCRs provided enough amplification product for cloning and sequencing. The primers used are listed in Table 1.

**TABLE 1:** Primer sequences for RLM-RACE

Primer	Sequence (5′–3′)	Target
5′-RACE outer forward	GCT GAT GGC GAT GAA TGA ACA CTG	5′ adapter
5′-RACE inner forward	CGC GGA TCC GAA CAC TGC GTT TGC TGG CTT TGA TG	5′ adapter
5′-RACE inner reverse	TGA ATT AGG AGG CGA CCG TA	OvHV-2 specific
5′-RACE outer reverse	AGA TTG CCA TCT GGG AGG TT	OvHV-2 specific
3′-RACE outer forward	TAC GGT CGC CTC CTA ATT CA	OvHV-2 specific
3′-RACE inner forward	GAT GGC AAT CTG CAA CCT TA	OvHV-2 specific
3′-RACE inner reverse	CGC GGA TCC GAA TTA ATA CGA CTC ACT ATA GG	3′ adapter
3′-RACE outer reverse	GCG AGC AGA ATT AAT ACG ACT	3′ adapter

**(ii) Cloning of the Ov8.25 locus into pEGFP N3.** The newly determined Ov8.25 locus was amplified by bracketing PCR primers (Table 2), which were supplemented with specific restriction enzyme digestion sites for later orientation-dependent subcloning. The ensuing 750-bp PCR product was initially cloned into the TOPO TA vector. After verifying its desired sequence, the desired fragment was excised from the TOPO vector by double digestion with NheI and EagI, whereas the pEGFP vector was opened by NheI and NotI digestion. After agarose gel electrophoresis, the desired fragments were cut out, pooled, and liquified for in-gel ligation using 1 μl of the vector (gel), 4 μl of the DNA fragment/insert (gel), 1 μl T4 DNA ligase, 2.5 μl T4 DNA buffer, and nuclease-free H_2_O to give a final volume of 26 μl. Sequencing of the ensuing cloned construct confirmed that the Ov8.25 locus was now in the pEGFP N3 vector and under the control of the cmvIE promoter.

**TABLE 2:** Primer sequences for cloning into the pEGFP N3 expression vector and transient expression

Primer	Sequence (5′–3′)^*a*^	Target
Forward Ov8.25 locus	*tcag****g***/***ctagc***AGTAAGCACACAGAAGGGAG	5′ of the 8.25 locus with an NheI site
Reverse Ov8.25 locus	*ggac****c***/***ggccg***GGACACTCATATTAATTAAAACATTTATTTAC	3′ of the 8.25 locus with an EagI site
Forward Ov8.25 intron	CCTGCTCATAATTATCTTTGCG	5′ of the first intron
Reverse Ov8.25 intron	TCCTCATCCTCATCTCTCTTCTT	3′ of the last intron

a Non-OvHV-2 sequences are in lowercase and italic type. Restriction enzyme sites are indicated in boldface type.

**(iii) Synthetic constructs.** Synthetic DNA sequences were obtained from GenScript (Piscataway, NJ, USA). Gateway technology (Invitrogen/Thermo Fisher Scientific) was used to transfer the synthetic constructs into pHSV amplicon vectors, whose design was described previously (41). A summary of the constructs is provided in Table 3.

**TABLE 3:** Overview of synthetic constructs

Designation^*a*^	Description	Purpose
Synthetic Ov8.25 locus	Reverse translated from the native amino acid sequence as determined from a Swiss cow with MCF, starting from the M codon of the predicted protein and ending prior to the stop codon; exon sequences codon optimized for translation in bovine cells; unaltered intron sequences; additional 5′ and 3′ nucleotides to provide restriction enzyme sites for orientation-dependent subcloning	Transient expression of an EYFP fusion protein; translated from mRNA after splicing
M-less Ov8.25	Same as synthetic Ov8.25 locus but with each ATG in the exons of ORF1 replaced by a stop codon; intron sequences remained native	Transient expression of EYFP without preceding pOv8.25; translated from mRNA after splicing
M-less intron 1 Ov8.25	Shorter version of the M-less Ov8.25 construct, flanking intron 1	Confirmation of splice sites
M-less intron 2 Ov8.25	Similar to the M-less intron 1 Ov8.25 construct but flanking intron 2	Confirmation of splice sites
Ov8.25 CDS	Intronless coding sequences of pOv8.25 without a stop codon; codon optimized for translation in bovine cells	Phenotype of the EYFP fusion protein in the absence of splicing

a CDS, coding DNA sequence.

### Cell culture.

Vero 2-2 cells are derived from an African green monkey Vero kidney epithelial cell line by incorporating a plasmid containing the IE2 (ICP27) gene and promoter into the genome. Vero 2-2 cells were cultured in Dulbecco’s modified Eagle’s medium (DMEM; Life Technologies) containing 10% heat-inactivated fetal calf serum (FCS) and Geneticin (500 μg/ml). Cells were cultured at 37°C in a humidified atmosphere containing 5% CO_2_.

HEK 293T cells were obtained from the ATCC and are human embryonic kidney cells that contain adenoviral and simian virus 40 (SV40) DNA sequences, which are particularly suitable for transient-expression assays. These cells were also kept in DMEM, propagated with FCS, and kept in 5% CO_2_.

*Theileria parva*-transformed bovine T lymphocytes (a generous gift from the late Dirk Dobbelaere, Institute of Molecular Parasitology, University of Bern) were cultured in RPMI containing 10% heat-inactivated fetal bovine serum (FBS) (catalogue number 10500-064; Gibco), 1% penicillin-streptomycin, and 0.001% β-mercaptoethanol (catalogue number 60242; Sigma-Aldrich). Cells were cultured at 37°C in a humidified atmosphere containing 5% CO_2_. Tp951-f53 is a cell line clone of PBMCs of animal 951 that were infected with *T. parva* Marikebuni (CTVM stabilate 72) of genotype F53 and cloned by limiting dilution. Tp951-f31 is a cell line clone of PBMCs of animal 951 that were infected with *T. parva* Marikebuni (CTVM stabilate 72) of genotype F31 and cloned by limiting dilution.

### Transient expression.

For transient expression, cloned DNA was transfected using Lipofectamine LTX into either HEK 293T or Vero 2-2 cells. Forty-eight hours after transfection, total RNA was extracted using the RNeasy minikit (Qiagen, Hombrechtikon, Switzerland). The extracted RNA was reverse transcribed using the reverse transcription protocol from Promega. Phusion high-fidelity DNA polymerase (Finnzymes, Thermo Fisher Scientific, Reinach, Switzerland) was used to amplify the cDNA by PCR, using primers (listed in Table 2) that bracketed the introns of interest. The amplified PCR products were then run on a 2% agarose gel (NEEO ultraquality agarose; Roth AG, Arlesheim, Switzerland).

### Polyacrylamide gel electrophoresis and Western transfer.

Twenty-four hours after transfection, the cells were washed once with phosphate-buffered saline (PBS) before 160 μl of immunoprecipitation (IP) buffer (1% Triton X-100, 20 mM Tris, 50 mM NaCl, 50 mM NaF, 150 mM Na_4_P_2_O_7_, 10 mM EDTA) was added. The cells were then scraped off and transferred into a 1.5-ml Eppendorf tube to be incubated on ice for 15 min. The lysate was then centrifuged in an Eppendorf centrifuge for 15 min at 13,000 rpm at 4°C. The soluble (supernatant) and insoluble (cell pellet) fractions were separated in two tubes. The cell pellet was resuspended in 84 μl of PBS and mixed with 16 μl of 6× Laemmli buffer, whereas 33 μl of 6× Laemmli buffer was added to the supernatant and boiled for 10 min at 95°C. The protein lysates were run in 15% polyacrylamide gels at 100 V for 2 h before being transferred onto polyvinylidene difluoride (PVDF) membranes (Bio-Rad) at 100 V for 1.5 h.

### Fluorescence microscopy and confocal microscopy.

Separate dishes of transfected cells were incubated at 37°C for 6, 24, or 48 h. In order to stain mitochondria, after each incubation time, cells were washed once with PBS before MitoTracker deep red (Invitrogen) diluted in culture medium (final concentration of 300 μM) was added to the respective wells, and the cells were incubated for 15 min at 37°C. In order to stain the ER and Golgi apparatus of transfected cells, cells were fixed with 4% paraformaldehyde (PFA) for 25 min, followed by permeabilization with 0.1% Triton X-100 for 2 min. Subsequently, cells were stained with ConA (20 μg/ml) and WGA (5 μg/ml) for 25 min at room temperature (RT). Stained cells were washed once with PBS and once with H_2_O. Finally, a drop of Roti-Mount FluorCare DAPI (4′,6-diamidino-2-phenylindole) was overlaid, and the cells were covered with a glass coverslip before being analyzed using a confocal microscope at wavelengths of 488 nm for EYFP, 594 nm for ConA, 594 nm for WGA, and 633 nm for MitoTracker deep red. Images were taken and analyzed using Imaris software. To quantify the amount of colocalization, a previously established method (Manders overlap coefficient) was used (42).

### Fluorescent stains.

The following fluorescent stains were used: Roti-Mount FluorCare DAPI (Carl Roth GmbH, Karlsruhe, Germany), MitoTracker deep red (633 nm; Invitrogen, Carlsbad, CA, USA), ConA-Alexa 594, WGA-Alexa 594, fluorescein isothiocyanate (FITC)-annexin V (AV) (Abcam, Cambridge, UK), and propidium iodide (PI) (Sigma-Aldrich, St. Louis, MO, USA).

### Annexin/PI staining.

Vero 2-2 cells were seeded in 6-well plates at 90% confluence. The next day, cells were either left untransfected or transfected with the respective plasmids. After either 48 h or 72 h, cells were harvested and washed once with PBS. A working dilution (100 μg/ml) of PI (Sigma-Aldrich Chemie GmbH, Buchs SG, Switzerland) was prepared. Next, 2 μl of FITC-annexin V (Abcam, Cambridge, UK) and 1 μl of PI (from the working dilution) were added to 100 μl of 1× annexin-binding buffer. Washed cells were resuspended in 100 μl of 1× annexin-binding buffer and incubated for 15 min in the dark. The cells were then filtered through a polystyrene round-bottom FACS tube with a cell strainer cap (BD Falcon), and data were acquired using flow cytometry. Three different channels were used: FL1 (EYFP), FL4 (PI), and FL6 (Cy5). The acquired data were analyzed using Kaluza (Beckman Coulter).

### Purified antibodies against Ov8.25 protein.

Rabbit antipeptide serum was commercially produced and purified by GenicBio Limited, Shanghai, China. The immunizing synthetic peptide (KKKDKKRDEDEDEDEDE-C) was conjugated to keyhole limpet hemocyanin (KLH). Specific antibodies were purified from the resulting antiserum using peptide-specific affinity.

Preimmune serum (2 ml), purified antibodies (4 mg), as well as 10 mg of unconjugated peptide were provided in a lyophilized form.

### Immunohistochemistry.

Due to the scarcity of the disease, fresh tissues from recent MCF cases were unavailable. As an alternative, we took advantage of an existing collection of formaldehyde-fixed, paraffin-embedded brain tissues from suspected bovine spongiform encephalitis (BSE) cases, which, however, had been deemed BSE negative but whose pathological diagnosis was encephalitis. One of these cases (case 44282 from 2010 [thalamus]) had been deemed OvHV-2 positive by PCR, whereas two other cases (case 33181 from 2001 [brain stem] and case 26731 from 1998 [brain stem]) had been deemed negative for OvHV-2. In the fourth case (case 51194 from 2018 [brain stem]), listeriosis had been diagnosed, but OvHV-2 was not formally excluded in fresh tissue. The brain tissues were cut into 3-μm sections. After antigen retrieval by boiling the samples for 20 min in a microwave in the presence of antigen retrieval buffer (Lsab; Dako), the sections were incubated overnight at 4°C with either purified rabbit anti-pOv8.25 serum or peptide-saturated antiserum. Subsequently, the reactions were made visible using a streptavidin-biotin complex method (Lsab; Dako) and AEC (3-amino-9-ethylcarbazole) as the chromogen.

## References

[1] Li H, Cunha CW, Taus NS, Knowles DP 2014 Malignant catarrhal fever: inching toward understanding. Annu Rev Anim Biosci 2:209–233. doi:10.1146/annurev-animal-022513-114156. 25384141

[2] Bedelian C, Nkedianye D, Herrero M 2007 Maasai perception of the impact and incidence of malignant catarrhal fever (MCF) in southern Kenya. Prev Vet Med 78:296–316. doi:10.1016/j.prevetmed.2006.10.012. 17123651

[3] Whitaker KA, Wessels ME, Campbell I, Russell GC 2007 Outbreak of wildebeest-associated malignant catarrhal fever in Ankole cattle. Vet Rec 161:692–695. doi:10.1136/vr.161.20.692. 18024925

[4] Meteyer CU, Gonzales BJ, Heuschele WP, Howard EB 1989 Epidemiologic and pathologic aspects of an epizootic of malignant catarrhal fever in exotic hoofstock. J Wildl Dis 25:280–286. doi:10.7589/0090-3558-25.2.280. 2716112

[5] Castro AE, Ramsay EC, Dotson JF, Schramke ML, Kocan AA, Whitenack DL 1984 Characteristics of the herpesvirus of malignant catarrhal fever isolated from captive wildebeest calves. Am J Vet Res 45:409–415. 6324620

[6] Muller-Doblies UU, Egli J, Li H, Braun U, Ackermann M 2001 Malignant catarrhal fever in Switzerland. 1. Epidemiology. Schweiz Arch Tierheilkd 143:173–183. (In German.) 11344942

[7] Li H, Cunha CW, Abbitt B, deMaar TW, Lenz SD, Hayes JR, Taus NS 2013 Goats are a potential reservoir for the herpesvirus (MCFV-WTD), causing malignant catarrhal fever in deer. J Zoo Wildl Med 44:484–486. doi:10.1638/2012-0087R.1. 23805572

[8] Russell GC, Stewart JP, Haig DM 2009 Malignant catarrhal fever: a review. Vet J 179:324–335. doi:10.1016/j.tvjl.2007.11.007. 18760944

[9] Ackermann M 2006 Pathogenesis of gammaherpesvirus infections. Vet Microbiol 113:211–222. doi:10.1016/j.vetmic.2005.11.008. 16332416

[10] Reid HW, Buxton D, Berrie E, Pow I, Finlayson J 1984 Malignant catarrhal fever. Vet Rec 114:581–583. doi:10.1136/vr.114.24.581. 6431686

[11] Albini S, Zimmermann W, Neff F, Ehlers B, Hani H, Li H, Hussy D, Casura C, Engels M, Ackermann M 2003 Porcine malignant catarrhal fever: diagnostic findings and first detection of the pathogenic agent in diseased swine in Switzerland. Schweiz Arch Tierheilkd 145:61–68. (In German.) doi:10.1024/0036-7281.145.2.61. 12649951

[12] Ackermann M 2005 Virus in sheep’s skin. Schweiz Arch Tierheilkd 147:155–164. (In German.) doi:10.1024/0036-7281.147.4.155. 15861922

[13] Sood R, Hemadri D, Bhatia S 2013 Sheep associated malignant catarrhal fever: an emerging disease of bovids in India. Indian J Virol 24:321–331. doi:10.1007/s13337-013-0163-y. 24426294 PMC3832689

[14] Stahel AB, Baggenstos R, Engels M, Friess M, Ackermann M 2013 Two different macaviruses, ovine herpesvirus-2 and caprine herpesvirus-2, behave differently in water buffaloes than in cattle or in their respective reservoir species. PLoS One 8:e83695. doi:10.1371/journal.pone.0083695. 24386255 PMC3873940

[15] Lapp S, Forster C, Kummrow M, Wohlsein P, Haist V 2015 Malignant catarrhal fever in a Vietnamese pot-bellied pig. A potential threat to pigs in mixed-species exhibits? Tierarztl Prax Ausg G Grosstiere Nutztiere 43:165–168. doi:10.15653/TPG-140494. 25947878

[16] Haigh JC, Mackintosh C, Griffin F 2002 Viral, parasitic and prion diseases of farmed deer and bison. Rev Sci Tech 21:219–248. doi:10.20506/rst.21.2.1331. 11974612

[17] O’Toole D, Li H, Sourk C, Montgomery DL, Crawford TB 2002 Malignant catarrhal fever in a bison (Bison bison) feedlot, 1993–2000. J Vet Diagn Invest 14:183–193. doi:10.1177/104063870201400301. 12033673

[18] Muller-Doblies UU, Egli J, Hauser B, Li H, Strasser M, Ehrensperger F, Braun U, Ackermann M 2001 Malignant catarrhal fever in Switzerland. 2. Evaluation of the diagnosis. Schweiz Arch Tierheilkd 143:581–591. (In German.) 11776716

[19] O’Toole D, Li H 2014 The pathology of malignant catarrhal fever, with an emphasis on ovine herpesvirus 2. Vet Pathol 51:437–452. doi:10.1177/0300985813520435. 24503439

[20] Palmeira L, Sorel O, Van Campe W, Boudry C, Roels S, Myster F, Reschner A, Coulie PG, Kerkhofs P, Vanderplasschen A, Dewals BG 2013 An essential role for gamma-herpesvirus latency-associated nuclear antigen homolog in an acute lymphoproliferative disease of cattle. Proc Natl Acad Sci U S A 110:E1933–E1942. doi:10.1073/pnas.1216531110. 23630278 PMC3666693

[21] Meier-Trummer CS, Rehrauer H, Franchini M, Patrignani A, Wagner U, Ackermann M 2009 Malignant catarrhal fever of cattle is associated with low abundance of IL-2 transcript and a predominantly latent profile of ovine herpesvirus 2 gene expression. PLoS One 4:e6265. doi:10.1371/journal.pone.0006265. 19603070 PMC2705673

[22] Meier-Trummer CS, Tobler K, Hilbe M, Stewart JP, Hart J, Campbell I, Haig DM, Glauser DL, Ehrensperger F, Ackermann M 2009 Ovine herpesvirus 2 structural proteins in epithelial cells and M-cells of the appendix in rabbits with malignant catarrhal fever. Vet Microbiol 137:235–242. doi:10.1016/j.vetmic.2009.01.030. 19249164

[23] Nelson DD, Taus NS, Schneider DA, Cunha CW, Davis WC, Brown WC, Li H, O’Toole D, Oaks JL 2013 Fibroblasts express OvHV-2 capsid protein in vasculitis lesions of American bison (Bison bison) with experimental sheep-associated malignant catarrhal fever. Vet Microbiol 166:486–492. doi:10.1016/j.vetmic.2013.07.021. 23953727

[24] Mushi EZ, Rurangirwa FR 1981 Epidemiology of bovine malignant catarrhal fevers, a review. Vet Res Commun 5:127–142. doi:10.1007/bf02214977. 7048724

[25] Hussy D, Stauber N, Leutenegger CM, Rieder S, Ackermann M 2001 Quantitative fluorogenic PCR assay for measuring ovine herpesvirus 2 replication in sheep. Clin Diagn Lab Immunol 8:123–128. doi:10.1128/CDLI.8.1.123-128.2001. 11139205 PMC96020

[26] Li H, Taus NS, Lewis GS, Kim O, Traul DL, Crawford TB 2004 Shedding of ovine herpesvirus 2 in sheep nasal secretions: the predominant mode for transmission. J Clin Microbiol 42:5558–5564. doi:10.1128/JCM.42.12.5558-5564.2004. 15583281 PMC535255

[27] Dobbelaere DA, Coquerelle TM, Roditi IJ, Eichhorn M, Williams RO 1988 Theileria parva infection induces autocrine growth of bovine lymphocytes. Proc Natl Acad Sci U S A 85:4730–4734. doi:10.1073/pnas.85.13.4730. 3133661 PMC280509

[28] Davison AJ, Eberle R, Ehlers B, Hayward GS, McGeoch DJ, Minson AC, Pellett PE, Roizman B, Studdert MJ, Thiry E 2009 The order Herpesvirales. Arch Virol 154:171–177. doi:10.1007/s00705-008-0278-4. 19066710 PMC3552636

[29] Ensser A, Pflanz R, Fleckenstein B 1997 Primary structure of the alcelaphine herpesvirus 1 genome. J Virol 71:6517–6525. doi:10.1128/JVI.71.9.6517-6525.1997. 9261371 PMC191927

[30] Hart J, Ackermann M, Jayawardane G, Russell G, Haig DM, Reid H, Stewart JP 2007 Complete sequence and analysis of the ovine herpesvirus 2 genome. J Gen Virol 88:28–39. doi:10.1099/vir.0.82284-0. 17170433

[31] Taus NS, Herndon DR, Traul DL, Stewart JP, Ackermann M, Li H, Knowles DP, Lewis GS, Brayton KA 2007 Comparison of ovine herpesvirus 2 genomes isolated from domestic sheep (Ovis aries) and a clinically affected cow (Bos bovis). J Gen Virol 88:40–45. doi:10.1099/vir.0.82285-0. 17170434

[32] Jayawardane G, Russell GC, Thomson J, Deane D, Cox H, Gatherer D, Ackermann M, Haig DM, Stewart JP 2008 A captured viral interleukin 10 gene with cellular exon structure. J Gen Virol 89:2447–2455. doi:10.1099/vir.0.2008/001743-0. 18796712

[33] Li H, Cunha CW, Taus NS 2011 Malignant catarrhal fever: understanding molecular diagnostics in context of epidemiology. Int J Mol Sci 12:6881–6893. doi:10.3390/ijms12106881. 22072925 PMC3211016

[34] Sorel O, Chen T, Myster F, Javaux J, Vanderplasschen A, Dewals BG 2017 Macavirus latency-associated protein evades immune detection through regulation of protein synthesis in cis depending upon its glycin/glutamate-rich domain. PLoS Pathog 13:e1006691. doi:10.1371/journal.ppat.1006691. 29059246 PMC5695634

[35] Nightingale K, Dry I, Hopkins J, Dalziel R 2019 Regulation of Ov2 by virus encoded microRNAs. Vet Res Commun 43:99–104. doi:10.1007/s11259-019-09749-9. 30888610 PMC6525144

[36] Dry I, Nightingale K, Ferguson J, Hopkins J, Dalziel R 2019 Ov2 is a modulator of OvHV-2 RTA mediated gene expression. Vet Res Commun 43:91–97. doi:10.1007/s11259-019-09748-w. 30900113 PMC6525121

[37] Bartley K, Deane D, Percival A, Dry IR, Grant DM, Inglis NF, McLean K, Manson ED, Imrie LH, Haig DM, Lankester F, Russell GC 2014 Identification of immuno-reactive capsid proteins of malignant catarrhal fever viruses. Vet Microbiol 173:17–26. doi:10.1016/j.vetmic.2014.07.004. 25091530

[38] Dewals B, Boudry C, Farnir F, Drion PV, Vanderplasschen A 2008 Malignant catarrhal fever induced by alcelaphine herpesvirus 1 is associated with proliferation of CD8+ T cells supporting a latent infection. PLoS One 3:e1627. doi:10.1371/journal.pone.0001627. 18286184 PMC2229840

[39] Bridgen A, Munro R, Reid HW 1992 The detection of alcelaphine herpesvirus-1 DNA by in situ hybridization of tissues from rabbits affected with malignant catarrhal fever. J Comp Pathol 106:351–359. doi:10.1016/0021-9975(92)90021-l. 1322946

[40] Rossiter PB 1980 A lack of readily demonstrable virus antigens in the tissues of rabbits and cattle infected with malignant catarrhal fever virus. Br Vet J 136:478–483. doi:10.1016/s0007-1935(17)32190-5. 6784880

[41] Meier AF, Laimbacher AS, Ackermann M 2016 Polycistronic herpesvirus amplicon vectors for veterinary vaccine development. Methods Mol Biol 1349:201–224. doi:10.1007/978-1-4939-3008-1_13. 26458838

[42] Dunn KW, Kamocka MM, McDonald JH 2011 A practical guide to evaluating colocalization in biological microscopy. Am J Physiol Cell Physiol 300:C723–C742. doi:10.1152/ajpcell.00462.2010. 21209361 PMC3074624

[43] Omasits U, Ahrens CH, Muller S, Wollscheid B 2014 Protter: interactive protein feature visualization and integration with experimental proteomic data. Bioinformatics 30:884–886. doi:10.1093/bioinformatics/btt607. 24162465

